# The Role of Extracellular HSP70 in the Function of Tumor-Associated Immune Cells

**DOI:** 10.3390/cancers13184721

**Published:** 2021-09-21

**Authors:** Manuel Linder, Elke Pogge von Strandmann

**Affiliations:** Institute for Tumor Immunology, Clinic for Hematology, Immunology, and Oncology, Philipps University of Marburg, Hans-Meerwein-Strasse 3, 35043 Marburg, Germany; manuel.linder@staff.uni-marburg.de

**Keywords:** HSP70, extracellular vesicles, tumor microenvironment, cancer, immune modulation

## Abstract

**Simple Summary:**

The intracellular heat shock protein 70 (HSP70) is essential for cells to respond to stress, for instance, by refolding damaged proteins or inhibiting apoptosis. However, in cancer, HSP70 is overexpressed and can translocate to the extracellular milieu, where it emerged as an important modulator of tumor-associated immune cells. By targeting the tumor microenvironment (TME) through different mechanisms, extracellular HSP70 can trigger pro- or anti-tumorigenic responses. Therefore, understanding the pathways and their consequences is crucial for therapeutically targeting cancer and its surrounding microenvironment. In this review, we summarize current knowledge on the translocation of extracellular HSP70. We further elucidate its functions within the TME and provide an overview of potential therapeutic options.

**Abstract:**

Extracellular vesicles released by tumor cells (T-EVs) are known to contain danger-associated molecular patterns (DAMPs), which are released in response to cellular stress to alert the immune system to the dangerous cell. Part of this defense mechanism is the heat shock protein 70 (HSP70), and HSP70-positive T-EVs are known to trigger anti-tumor immune responses. Moreover, extracellular HSP70 acts as an immunogen that contributes to the cross-presentation of major histocompatibility complex (MHC) class I molecules. However, the release of DAMPs, including HSP70, may also induce chronic inflammation or suppress immune cell activity, promoting tumor growth. Here, we summarize the current knowledge on soluble, membrane-bound, and EV-associated HSP70 regarding their functions in regulating tumor-associated immune cells in the tumor microenvironment. The molecular mechanisms involved in the translocation of HSP70 to the plasma membrane of tumor cells and its release via exosomes or soluble proteins are summarized. Furthermore, perspectives for immunotherapies aimed to target HSP70 and its receptors for cancer treatment are discussed and presented.

## 1. Introduction

Heat shock protein 70 (HSP70/HSPA1A/HSP72) is a molecular chaperone and belongs to the HSP70 family. It is upregulated upon stress-stimuli, such as heat, exercise, or pathological stress. HSP70 comprises a plethora of crucial housekeeping and chaperone activities, including folding of newly synthesized proteins, refolding or disposal of damaged proteins, preventing protein aggregates, or translocating proteins to different compartments. In addition, HSP70 can directly inhibit apoptosis, thus protecting cells from stress-induced cell death [1]. However, in pathologies like cancer, upregulated HSP70 can lead to disease progression and therapy resistance [2]. The chaperone consists of three main domains, the N-terminal ATPase domain (NBD), the substrate-binding domain (SBD), and a C-terminal domain. The NBD is crucial for binding and hydrolyzing ATP to ADP, thus regulating the chaperone’s conformational state. In general, the binding of ATP leads to an open or low-affinity conformation allowing HSP70 to bind its substrates via its SBD. In the ADP state, the chaperone turns into a closed or high-affinity conformation in which the protein is further protected and processed. Importantly, all functions of HSP70 require different co-chaperones, such as BCl-2-associated anthanogene 3 and 4 (BAG3, BAG4) or carboxy-terminus of HSP70 interacting protein (CHIP), and is mainly mediated by the C-terminal region of the chaperone [1]. Initially, HSP70 was identified as an intracellular protein (iHSP70), but it can translocate to the extracellular milieu (exHSP70) under stressful conditions. This includes physiological stress (e.g., physical activity) and pathological stress (e.g., cancer) and is higher in the latter. Hereby, it is either associated with the plasma membrane (mHSP70), with extracellular vesicles (evHSP70), or secreted as soluble proteins (sHSP70) [2].

Extracellular vesicles (EVs) are nanoparticles secreted by virtually all cells under physiological and pathophysiological conditions. EVs differ in size, origin, and cargo and can be mainly subdivided into ectosomes and exosomes [3]. Ectosomes, also referred to as microvesicles, are generally described as particles with a size of 50–1000 nm, generated by a direct outward budding of the plasma membrane. In contrast, exosomes are small EVs with a size between 40 and 200 nm and are generated along an endosomal pathway by double invagination of the plasma membrane (Box 1). EVs contain a plethora of molecules, including proteins, lipids, and nucleic acids, usually reflecting the cell of origin [3]. In the past, it has been speculated that they function as trash bins to remove unwanted cellular components; however, increasing evidence demonstrates crucial functions as an intercellular communication system [3,4,5].

Box 1 **Biology and function of exosomes**

Exosomes are small extracellular vesicles (40–200 nm; small EVs), which are produced by a double invagination of the plasma membrane. In particular, endocytosis leads to the generation of early sorting endosomes (ESE), containing factors from the cell membrane and the extracellular milieu. After maturation to late sorting endosomes (LSE), a second invagination of the membrane occurs, leading to the formation of multivesicular bodies (MVB) containing future exosomes. During MVB formation, cargo is specifically sorted to the vesicles by several distinct mechanisms. The endosomal sorting complexes required for transport (ESCRT) machinery is widely believed to play a role in the sorting process; however, specific loading mechanisms remain largely unknown and need to be elucidated. Typical exosomal markers often include ESCRT components, such as Alix or TSG101. Interestingly, HSP70 is also described as an exosome marker; however, its expression is not specific to the small EVs. The cargo can include various proteins, lipids, or nucleic acids, indicating a multitude of distinct functions. After the release, exosomes can interact with target cells by binding to specific receptors, fusion with the plasma membrane, or endocytosis. Since they are critical players of intercellular communication in physiological and pathological conditions, the research interest significantly increased in the last decade [3,4].

In this review, we summarize the current knowledge on the HSP70 translocation to the extracellular milieu, either in association with the plasma membrane, extracellular vesicles, or as soluble extracellular proteins. Moreover, we outline the functions of exHSP70, especially its ability to modulate the immune system in cancer. We address possible interactions of the chaperone with the tumor microenvironment. Finally, we provide an overview of the potential therapeutic options regarding exHSP70.

## 2. Translocation of exHSP70

HSP70 has several functions, including folding newly synthesized proteins, regulating protein activity, or preventing aggregation, indicating a cytosolic localization of the chaperone [1]. However, HSP70 has also been found on the plasma membrane, associated with EVs, or secreted as free soluble protein [6,7,8]. Here, the translocation was shown to occur independently of the physiological state, although stress, such as in cancer, drastically increases the extracellular localization [8,9].

In the classical secretory pathway, proteins synthesized by ribosomes are released into the lumen of the endoplasmic reticulum (ER), where chaperones assist and control the proper protein folding. Correctly folded proteins subsequently enter the Golgi apparatus and are eventually secreted by transport vesicles. This pathway usually requires a short peptide sequence that targets proteins for secretion [10]. HSP70 lacks such a specific signal peptide indicating different mechanisms for its translocation [11].

### 2.1. Membrane HSP70 (mHSP70)

In accordance, Broquet and colleagues reported an unhindered translocation of HSP70 to the plasma membrane after treatment with the classical secretory pathway inhibitors brefeldin A or monensin [12]. Brefeldin A is an antiviral lactone compound blocking the anterograde transport from the ER to the Golgi. In contrast, monensin is a polyether antibiotic acting as an ionophore, inhibiting the transport from the Golgi apparatus [13]. This study further revealed that mHSP70 preferentially localizes in lipid rafts, which are microdomains enriched in cholesterol, glycosphingolipids, and protein receptors. They typically form in the exoplasmic leaflet of the Golgi apparatus and are also known as detergent-resistant microdomains (DRMs) due to their composition and resistance to non-ionic detergents [14]. Moreover, treatment with the drug methyl-β-cyclodextrin (MβCD), which degrades cholesterol and disrupts DRMs, resulted in a significant decrease in mHSP70, suggesting lipid raft-mediated translocation of HSP70 [12]. In accordance, Hunter and Levin showed HSP70 release in peripheral blood mononuclear cells (PBMCs), which could be blocked by MβCD but not by brefeldin A [15]. However, in contrast to the previous study, translocation was also partially inhibited by monensin. The authors postulated that the Golgi but not the ER might be important for HSP70 secretion or that monensin led to a disruption of the plasma membrane, ultimately inhibiting the transport [15]. It has also been reported that HSP70 can non-covalently bind to the lipid raft component globotriaosylceramide (Gb3), further supporting the role of lipid rafts in the translocation of the chaperone [16,17]. Therefore, HSP70 could be associated with Gb3 and subsequently secreted and recruited to lipid rafts. Another mechanism of membrane integration by HSP70 could be a direct interaction with the plasma membrane, as suggested by its ability, to bind to phosphatidylserine (PS), integrate into membranes, or form ionic channels [7,18,19,20,21]. It has been hypothesized that HSP70 translocates to the membrane after oligomerization, where it binds to PS [7]. Spontaneous flipping allows PS to reach the outer membrane layer, leading to the integration of the chaperone. It is further described that the return of PS into the cytosolic layer does not affect the integration of HSP70 [7].

### 2.2. EV-Associated HSP70 (evHSP70)

In 2005, Lancaster and co-workers presented another mechanism for the translocation of HSP70 [6]. They reported that HSP70 is released by exosomes in PBMCs independently of stress. Neither brefeldin A nor MβCD were able to inhibit the transport, indicating an endosomal-mediated secretion mechanism. It was further postulated that the controversial results of previous studies in PBMCs from Hunter and Levin could be due to an insufficient concentration of the inhibitor MβCD [6,15]. However, distinct exocytosis processes could occur in different and even within the same cell types. It can be hypothesized that translocation of HSP70 into or onto exosomes could eventuate after the chaperones are localized in DRM. Lipid raft-mediated endocytosis could then lead to the formation of endosomes, further maturing into multivesicular bodies (MVB) containing future exosomes. Many other reports have confirmed exosomal secretion of HSP70; however, the mechanism of sorting the chaperone to the vesicles is still elusive [7,22,23,24]. In general, posttranslational modifications, including ubiquitination, phosphorylation, or sumoylation, have been indicated to play a role in cargo sorting into exosomes [25,26,27,28]. Interestingly, HSP70 was found to be ubiquitinated by the co-chaperone CHIP, mainly presumed to mark the protein for degradation [29,30]. However, Jiang and colleagues showed that ubiquitylation of the constitutive isoform HSC70 by CHIP did not lead to an increased degradation [31]. This is in line with a report displaying that crucial amounts of secreted HSP70 in A431 cells are ubiquitinylated [32]. In contrast, acetylation of the HSP70 family member high glucose-regulated protein 78 (GRP78) was shown to inhibit exosomal secretion through interaction with the phosphoinositide-3 kinase VPS34 [33]. Acetylated HSP70 was also reported to bind to VPS34 [34]. Hereby, autophagosomal stress led to disruption of the interaction of histone deacetylase 6 (HDAC6) with the chaperone, resulting in increased acetylation. Interestingly, oligomerization, which was shown to play a role in membrane integration of HSP70, was indicated to be preferentially loaded into exosomes [7,35]. This is supported by studies of Nimmervoll and co-workers, demonstrating mHSP70-mediated clathrin-independent endocytosis, which was dependent on the oligomerization of the chaperone [36,37]. Therefore, HSP70 could be sorted into exosomes after integration into the membrane in an oligomeric form.

### 2.3. Soluble Extracellular HSP70 (sHSP70)

In addition to mHSP70 and evHSP70, the chaperone can also be found in a free soluble form, which was previously thought to result exclusively from passive release upon cell death [11]. In particular, necrosis rather than apoptosis was assumed to be responsible for the release of sHSP70 [38]. However, cell death accounts only for a minor fraction of sHSP70, and it is mainly released in an active manner [11]. Mambula and Calderwood postulated an endolysosomal route as a mechanism for solubleHSP70 (sHSP70), as the secretion correlated with the lysosomal marker lysosomal-associated membrane protein 1 (LAMP1) and could be inhibited by lysosomotropic compounds [39]. Moreover, they showed that HSP70 could enter lysosomes via ATP-binding cassette (ABC) family transporter proteins [39]. Interestingly, ABC transporters are also expressed on endosomes and exosomes, where one could postulate a possible way for HSP70 loading into exosomes. The secreted chaperone was also found to bind back to the plasma membrane, indicating an alternative route to the lipid raft-mediated pathway for membrane-associated HSP70 [39]. 

Another way of HSP70 release was demonstrated by Evdonin et al., showing the formation of secretory-like granules upon inhibition of phospholipase C [32]. Interestingly, this secretion could be blocked by brefeldin A, indicating an involvement of the classical secretory pathway [32,40]. This was contradictory to prior studies that demonstrated no inhibition of the secretory pathway by brefeldin A [6,12,15]. However, the authors suggested a time-dependent effect since previous studies evaluated the translocation of HSP70 at least 4 h after inducing stress or treatment with brefeldin A [40]. Therefore, it can be postulated that distinct cells potentially use different mechanisms, possibly depending on the stress level and exposure. 

**Figure 1:**
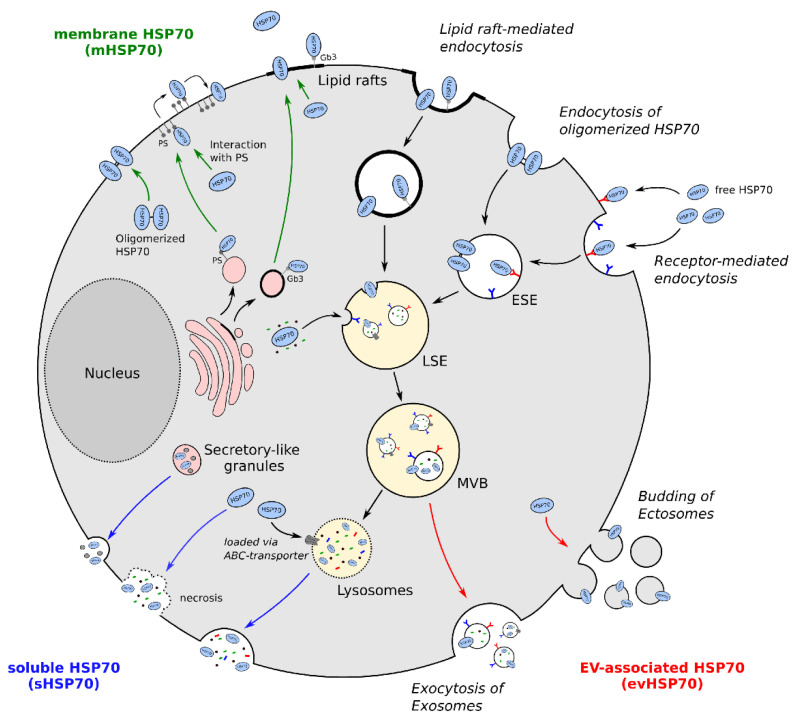
Translocation of extracellular HSP70 (exHSP70). Cellular stress leads to the translocation of HSP70 via different potential mechanisms. In particular, membrane-associated HSP70 (mHSP70, green) can be found in lipid rafts, mainly interacting with globotriaosylceramide 3 (Gb3). Moreover, HSP70 can interact with phosphatidylserine (PS), leading to its integration into the plasma membrane. Oligomerized HSP70 was also reported to integrate into the plasma membrane directly. HSP70 can also be released into or onto extracellular vesicles (evHSP70, red), by either budding of the plasma membrane (ectosomes) or by an endolysosomal pathway (exosomes). Soluble HSP70 (sHSP70, blue) can be secreted either passively during cell necrosis or actively via secretory granules or lysosomes. ESE: early sorting endosomes; LSE: late sorting endosomes; MVB: multivesicular bodies; EV: extracellular vesicles; PS: phosphatidylserine; Gb3: globotriaosylceramide 3; HSP70: heat shock protein 70.

Altogether, it can be concluded that translocation of HSP70 into the extracellular milieu is increased under stress conditions and that several pathways lead to either membrane-, EV-associated, or soluble HSP70 (Figure 1). Still, additional research is needed to further unravel the different mechanisms and their functional consequences. 

## 3. Role of exHSP70: Regulation of Immune Responses

The immune system is a complex network of biological processes protecting us from various pathogens and diseases. It is mainly divided into two groups, the innate immune system, which is defined by a non-specific and rapid response, and the adaptive immune system, which can respond and adapt to specific stress stimuli. A typical immune response can be divided into four phases: (I) Recognition of pathogen- or damage-associated molecular patterns (PAMPs/DAMPs) by innate immune cells resulting in phagocytosis, complement activation, and secretion of pro-inflammatory cytokines. (II) Released cytokines, such as IL-1, Il-12, or TNF-α, then trigger an acute inflammatory response helping to control the infection. (III) Meanwhile, antigen-presenting cells (APCs) activate naïve T-helper cells via their MHC class II and co-stimulatory signal molecules. (IV) Activated T-helper cells subsequently initiate the adaptive immune system, including a cell-mediated and humoral immune response [41]. 

In the case of inflammation, intracellular HSP70 expression is dramatically increased and exerts cytoprotective functions. This is achieved both through classical chaperone functions, such as refolding and repair of proteins, and by direct inhibition of apoptosis [42]. Since excessive apoptosis can lead to severe human inflammatory diseases (HIDs), HSP70 plays a crucial role in balancing the appropriate response to cell stress. However, in cancer, HSP70 is known to be overexpressed, upsetting the balance and increasing proliferation, invasiveness, and resistance of malignant cells [2]. As described in the previous chapter, HSP70 is known to translocate into the extracellular milieu primarily upon stress stimuli. Moreover, exHSP70 was shown to exert pro-inflammatory functions, leading to increased tissue damage, indicating a dual role of HSP70 [43]. In the following section, we provide an overview of the general mechanisms of exHSP70 in immunity and summarize current data of its functions towards immune cells in the tumor microenvironment. 

### 3.1. General Mechanisms of Immunomodulation by exHSP70

The role of HSP70 in immunity is still extensively discussed in the literature, and a plethora of distinct functions and mechanisms are described. It is associated with developing an innate and adaptive immune response, formation of memory cells, and termination of the immune response [2]. 

To exert specific immunomodulatory functions, proteins need to interact with immune cells. Here, membrane-bound and free HSP70 were both shown to bind to different immune cells, including macrophages, dendritic cells, and natural killer (NK) cells [44,45,46]. Moreover, in the early 2000s, Asea and colleagues demonstrated that exHSP70 specifically binds to monocytes, increasing the pro-inflammatory cytokines TNF-α, IL-6, and IL-1β [47]. This was postulated to be mediated by two distinct pathways: a CD14-dependent and a CD14-independent mechanism, with both being dependent on intracellular calcium. Consequently, exHSP70 binds monocytes, increasing the intracellular calcium flux and resulting in NF-κB-mediated transcription of pro-inflammatory cytokines [47]. Hence, the authors termed HSP70 a “chaperokine” [48]. This finding led to many publications displaying pro-inflammatory cytokine functions of HSP70 [49,50,51]. In addition to CD14, Toll-like receptors 2 and 4 (TLR2/TLR4) and CD40 were also described to mediate those effects, mainly via NF-κB and the MAPK pathway [49,51,52]. The fact that HSP70 could be released during necrotic cell death and subsequently induce an inflammatory response by TLR and being increasingly expressed in various inflammatory diseases emphasized its role as a DAMP [38,47,51].

However, shortly after the first publication, Gao and Tsan issued their concerns about the pro-inflammatory functions and postulated that endotoxin contamination and not HSP70 itself could trigger those effects [53]. They showed that highly purified recombinant HSP70 could not induce TNF-α in murine macrophages [53]. Moreover, endotoxin-free HSP70 was not able to mature dendritic cells, as described earlier [38,54]. Further studies from different groups supported the theory that most pro-inflammatory functions could be due to contamination [55,56,57,58]. Therefore, results obtained using recombinant HSP70, mainly produced in *Escherichia coli (E. coli)*, need to be interpreted with caution. In addition, studies with purified recombinant proteins are limited since the activity of HSP70-protein complexes is often overlooked. 

Fong and co-workers gave another explanation of the contradictory results. They showed that exHSP70 binds to sialic acid-binding immunoglobulin-type lectins (Siglecs), which are membrane proteins expressed on immune cells. In particular, exHSP70 can interact with the anti-inflammatory Siglec-5 as well as the pro-inflammatory Siglec-14. Different expression patterns of Siglecs in specific immune cell populations could therefore explain different inflammatory responses [59].

Interestingly, recent studies introduced another receptor that potentially induces pro-inflammatory functions [60,61]. Here, the authors showed that exHSP70 binds to the receptor for advanced glycation endproducts (RAGE), leading to extracellular signal-regulated kinases (ERK)-dependent activation of NF-κB. Apart from releasing pro-inflammatory cytokines, activated NF-κB also increased RAGE expression, resulting in a positive feedback loop [61]. 

In contrast, it was also reported that exHSP70 has rather anti-inflammatory than pro-inflammatory functions [62]. Studies showed that highly purified exHSP70 downregulated TNFα and IL-6 production in monocytes [63]. This was explained by upregulation of the heat shock factor 1 (HSF1), subsequently inhibiting NF-κB activation and directly binding the *TNFα* gene promotor [63,64]. Extracellular HSP70 was also shown to repress LPS-induced cytokines in rats [65]. In addition, in most neurological disorders, the chaperone was described to be primarily anti-inflammatory [66]. 

The function of HSP70 in the adaptive immune response is less controversial. It is widely accepted that the chaperone plays a significant role in the antigen-presenting process. Firstly, HSP70 can bind antigens either inside or outside of the cell [67]. The complex is then recognized by APCs via the CD91 or scavenger receptors, such as LOX-1 or SREC-1, resulting in endocytosis [68]. Inside of the APCs, HSP70 protects the antigen until it reaches the proteasome. Here, the antigen is released, processed, and transported to MHC class I molecules [69]. This unique cross-presentation finally leads to the activation of CD8^+^ T-cells. Moreover, the HSP70-antigen complex can be processed in the lysosome leading to the presentation of the antigen on MHC class II molecules, subsequently activating CD4^+^ T-cells [69]. The presentation itself was shown to be significantly enhanced by the HSP70-antigen complex compared to the antigen alone [70]. Therefore, HSP70 is essential for the transport, uptake, protection, and effective (cross-)presentation of antigens. 

Additionally, to the four phases of a typical immune response, the generation of immunological memory is crucial. Generally, the formation of B- and T-memory cells depends on the respective B- and T-cell receptors (BCR/TCR) and the MHC class II antigen complex [41]. In 2005, researchers of the University of Tuebingen showed enhanced activation of CD4^+^ memory T-cells by HSP70-peptide complexes compared to peptides alone [71]. This activation was shown to be dependent on CD91 and scavenger receptors [72]. Moreover, Wang and colleagues demonstrated a TCR- and MHC class II-independent mechanism of CD4^+^ memory T-cell formation [73]. Here, stress-induced dendritic cells upregulated intracellular and membrane-associated HSP70, which activates NF-κB via CD40, resulting in increased membrane-bound IL-15 expression. The IL-15 then activates the JAK3 and STAT5 pathway in CD4^+^ T-cells, upregulating CD40L, which can reactivate the DCs for a positive feedback loop and subsequently lead to the formation of CD4^+^ memory T-cells [73]. This was the first time researchers could demonstrate the formation of memory cells independent of the antigen. These results were also validated in vivo [74].

The last step of the immune response is the termination, which is vital to prevent tissue damage by excessive inflammation [75]. Overexpression of HSP70 on the surface of immune cells serves as a regulator for the termination by displaying a “death signal” [76]. HSP70 is hereby recognized by γδT killer cells, which subsequently terminate the cells [76]. Moreover, HSP70 was shown to prime γδT killer cells, leading to higher proliferation and killing [77]. This priming could be dependent on TLRs [78].

All in all, HSP70 is crucially involved in many aspects of immunity, including the development of the innate and adaptive immune response, the formation of memory cells, and the termination of the immune response. Hereby, the chaperone exhibit either pro- or anti-inflammatory functions, potentially depending on its location, cell type, and expression level. Several receptors are known to interact with exHSP70, such as CD40, TLR2, TLR4, CD14, RAGE, CD91, as well as different scavenger receptors, primarily exhibiting their function via the NF-κB or MAPK pathway.

### 3.2. The Role of exHSP70 in Immunomodulation of Cancer

The communication of cancer cells and immune cells is a crucial step in cancer progression. Therefore, understanding the mechanisms and consequences of the interaction is essential. A potential key player of the crosstalk in the tumor microenvironment may be HSP70, which is overexpressed in cancer compared to normal tissue [2,79,80,81,82]. The expression was shown to be correlated to tumor grade, therapy resistance, and worse overall survival [80,81,82,83,84,85]. In particular, HSP70 can inhibit the intrinsic and extrinsic apoptotic pathway and block oncogene-induced senescence, resulting in therapy resistance [2]. The chaperone was also reported to be translocated into the extracellular milieu in cancer, including as a membrane-bound or as an exosome secreted form [82,83,86,87,88,89]. Recently, Finkernagel and colleagues were able to identify HSP70 as a major constituent of ovarian cancer EVs and demonstrated a significant correlation of exHSP70 with patient survival [88]. Moreover, in a prospective clinical study of breast and lung cancer, exosomal HSP70 was correlated to metastasis and disease status. Here, the authors suggested HSP70-positive exosomes as a potential biomarker to predict tumor responses and clinical outcomes [82]. 

One candidate of the pro-tumorigenic effects of exHSP70 could be TLR4 and the subsequent PI3K/Akt pathway engagement. It is described that the pathway leads to IL-10 and galectin-1 production, resulting in an increase of matrix-metalloproteases 2 and 9 (MMP-9/MMP-2), finally enhancing tumor migration [90]. The induction of MMP-2 and MMP-9 by exosomal HSP70 was already reported in mesoangioblasts, where initiation occurred via TLR4 and CD91 in an autocrine fashion [91]. In addition, MMP-9 induction was also reported in monocytes. Extracellular HSP70 stimulated NF-κB and activating protein-1 (AP-1), enabling MMP-9 expression [92]. Interestingly, HSP70 was also shown to increase its own expression via the TLR4 by inactivating glycogen synthase kinase-3β (GSK-3β) via Akt signaling. Inactivation of GSK-3β then stimulated HSF1, finally inducing intracellular HSP70 [93]. It was also reported that tumor-derived exosomal HSP70 activated myeloid-derived suppressor cells (MDSC), enhancing tumor growth [94]. This is due to TLR2 activation and subsequent MyD88-dependent phosphorylation of the signal transducer and activator of transcription 3 (STAT3) [94,95]. STAT3 is known to be critically involved in tumor progression and generation of an immunosuppressive and therefore pro-tumorigenic environment [96,97]. Moreover, activation of TLR2 by exosomal HSP70 led to upregulation of IL-6, iNos, and Arg-1 [95]. Thereby, iNos enhances nitric oxide production, whereas Arg-1 leads to arginine depletion, both inhibiting T-cell proliferation and function [98,99]. MyD88-independent activation of TLR4 by exHSP70 was also described to facilitate cancer growth, potentially through the TRIF pathway [100]. 

The interaction of exHSP70 with TLR 2/4 was further demonstrated to activate neutrophils [88]. Klink and co-workers showed that activation of those receptors led to the production of reactive oxygen species (ROS) and the release of IL-8. This is associated with cancer progression since ROS can stimulate the expression of vascular endothelial growth factor (VEGF) and activate MMPs, thus enhancing tumor angiogenesis and metastasis [88,101]. Moreover, exHSP70-TLR2 interaction also leads to activation and pro-inflammatory cytokine production in neutrophils [102]. 

Additionally, the TLR4 pathway was suggested to play a role in chemotherapy resistance [103]. In ovarian cancer, TLR4 activation resulted in MyD88-dependent nuclear localization of NFκB, upregulating the production of IL-6 as well as the chemokines MCP-1 and GRO-α, which are all associated with tumor progression. Furthermore, the Akt pathway was activated, followed by enhanced expression of the anti-apoptotic protein XIAP, thus exhibiting resistance to chemotherapy [103,104]. In accordance, a recent study showed that transfer of exHSP70 via small EVs resulted in therapy resistance of breast cancer by increasing ROS production [105].

Epithelial to mesenchymal transition (EMT) is believed to be a key step of cancer cells to enable tumor invasion and metastasis. Li and colleagues showed that treating cells with tumor-derived exHSP70 resulted in the decrease of E-cadherin and the increase of α-SMA and, therefore, in the induction of EMT [106]. This was mediated by TLR2/4 and subsequent activation of the JNK1/2 and MAPK pathways [107]. 

**Figure 2:**
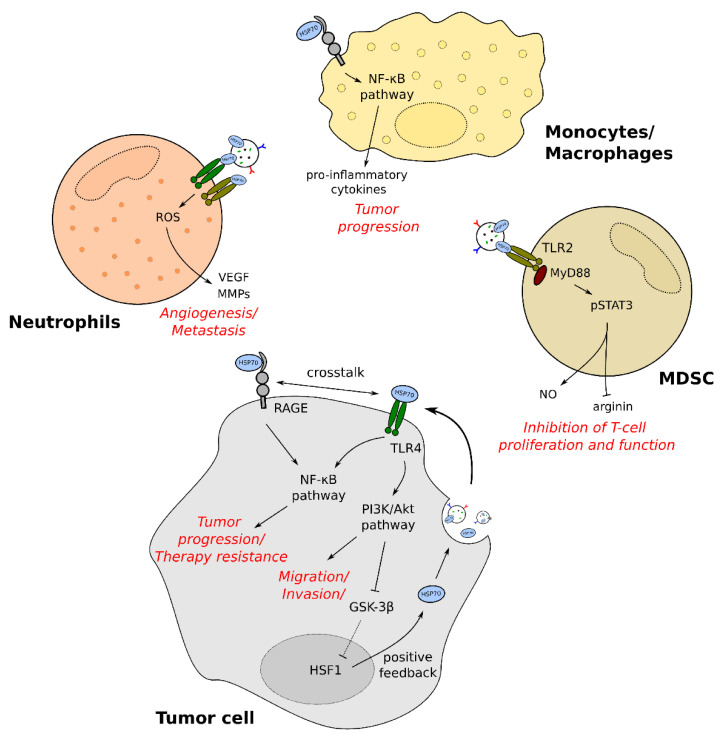
Pro-tumorigenic functions of exHSP70. MDSC: myeloid-derived suppressor cell; RAGE: receptor for advanced glycosylation endproducts; GSK-3β: glycogen synthase kinase-3β; NO: nitride oxide; TLR: Toll-like receptor; NFκB: nuclear factor kappa-light-chain-enhancer of activated B cells; HSF1: heat shock factor 1; VEGF: vasculogenic endothelial growth factor; pSTAT3: phosphorylated signal transducer and activator of transcription 3; ROS: reactive oxygen species; MyD88: myeloid differentiation primary response 88; PI3K: phosphoinositide 3-kinase; Akt: protein kinase B; MMP: matrix metalloproteinases; HSP70: heat shock protein 70.

Another target of exHSP70 is the receptor RAGE, which was shown to result in a pro-inflammatory response via ERK-dependent NF-κB activation [61]. Interestingly, RAGE was also found to be overexpressed in cancer, where it was correlated to tumor size and cancer stage [108,109]. Additionally, RAGE is expressed on immune cells, such as monocytes or macrophages, further extending possible targets for exHSP70 [110]. It is also described that RAGE interacts with TLR4 and that this crosstalk leads to MyD88-dependent activation of NF-κB [110,111] (Figure 2). 

**Figure 3:**
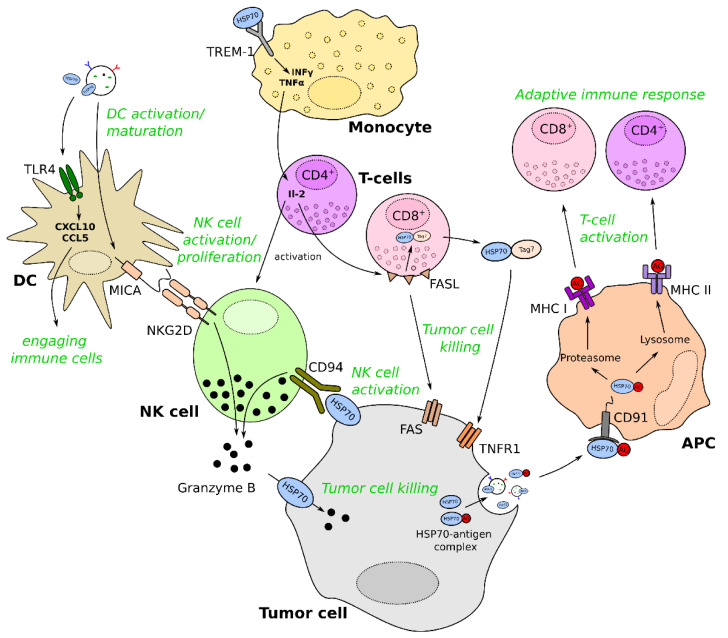
Anti-tumorigenic functions of exHSP70. FASL: Fas ligand; TNFR1: tumor necrosis factor receptor 1; APC: antigen-presenting cell; NK: natural killer; DC: dendritic cell; MHC: major histocompatibility complex; MICA: MHC class I polypeptide–related sequence A; TREM-1: triggering receptor expressed on myeloid cells 1; INF-γ: interferon-gamma; TNF-α: tumor necrosis factor-alpha; IL-2: interleukin-2; Tag7: peptidoglycan recognition protein; CXCL10: C-X-C motif chemokine ligand 10; CCL5: Chemokine (C-C motif) ligand 5; HSP70: heat shock protein 70.

Extracellular HSP70 can also exert anti-tumorigenic functions, which can be mediated by activating or priming NK-, dendritic- or T-cells (Figure 3). For instance, NK cells specifically interact with mHSP70 on tumor cells, probably via the C-type lectin receptor CD94 [112,113,114]. Moreover, it was described that exHSP70 could mature dendritic cells and increase the expression of the NK ligand MICA and the co-stimulatory molecules CD86 or CD40. Therefore, exHSP70 stimulates NK cells either directly via CD94 or indirectly via MICA, which binds the NK cell activating receptor NKG2D [87,115]. NK cells can then kill tumor cells in an NKG2D-dependent way or by increasing granzyme B release [115]. Interestingly, granzyme B was found to be incorporated in tumor cells by explicitly binding to mHSP70, which results in perforin-independent apoptosis of the cells [44]. Moreover, Sharapova and colleagues recently reported another possibility of NK cell activation while investigating a novel exHSP70 target receptor [116]. They showed that exHSP70 binds TREM-1 on monocytes, leading to the secretion of TNF-α and INF-γ. The cytokines then stimulate CD4^+^ T-cells to secrete IL-2, finally activating NK cells. Moreover, CD8^+^ T-cells are also activated by this mechanism and can kill tumor cells via FasL/Fas interaction [116]. Interestingly, FasL can trigger the secretion of an HSP70-Tag7 complex in T-cells. This complex further induces tumor cell lysis via the tumor necrosis factor receptor 1 (TNFR1) [117,118].

Dendritic cells can also be stimulated by binding to the TLR4. Activation of MyD88- and TRIF-dependent pathways result in the production of chemokines, such as CXCL10 or CCL5, engaging immune cells and inducing anti-tumor immunity [119]. 

Furthermore, exHSP70 was reported to inhibit the conversion of monocytes to a pro-tumor phenotype, which is potentially due to a diminished expression of pro-tumor cytokines, such as IL-10 and MCP-1 [120].

Finally, as described in the previous chapter, exHSP70 can induce an adaptive immune response by (cross-) presentation of antigens on MHC class I or II molecules. In particular, HSP70-antigen complexes are endocytosed by APCs after binding to CD91 or other scavenger receptors, where the antigen is processed and presented to T-cells [69].

Increasing evidence emphasizes exHSP70 as an important player in mediating immune responses in cancer by either exerting pro- or anti-tumorigenic functions. However, there is still much unknown or controversially described; thus, specific functions and their associated mechanism urgently need to be investigated. 

## 4. Therapeutic Potential of exHSP70

Since intracellular HSP70 is upregulated in cancer and elicits anti-apoptotic functions, it represents an important target for developing new therapeutics. Typical inhibitors are small molecules targeting various aspects of the HSP70 machinery [121]. These include ATPase and complex inhibitors as well as nucleotide-binding domain (NBD)- or substrate-binding domain (SDB)-targeting inhibitors [121]. Apart from small molecule inhibitors, Minnelide, a Triptolide derivative, has been shown to effectively downregulate HSP70 by inducing the miRNA miR-142–3p [122,123]. Additionally, Minnelide is currently tested in several phase I and II studies, including pancreatic cancer and acute myeloid leukemia [2]. Although targeting iHSP70 and therefore altering its expression or function also affects exHSP70, the linkages and consequences are not well understood.

### 4.1. HSP70 Antibody

The fact that mHSP70 is uniquely expressed on cancer cells compared to normal tissue makes it an excellent therapeutic target [124,125]. Gabrielle Multhoff’s group developed a specific IgG antibody directed against the exposed region of mHSP70 by immunizing mice with a 14-mer peptide (TKDNNLLGRFELSG, known as TKD) that encompasses the amino acids 450–463 of the chaperone [126,127]. This antibody, known as cmHsp70.1 mAb, was shown to bind specifically to mHSP70 in vitro and in vivo [127]. In addition, antibody-dependent cell-mediated cytotoxicity (ADCC), induced by the Fc-region of the antibody, was shown to significantly inhibit tumor growth in a preclinical study in mice [126]. However, IgG antibodies are inefficient in inducing ADCC, primarily via neutrophils, due to the binding of several inhibitory Fc-receptors [128]. Therefore, IgA antibodies should be considered an alternative since they efficiently induce ADCC by binding to the Fcα-receptor (CD89) on neutrophils, monocytes, and macrophages [129]. Furthermore, IgA antibodies do not activate the classical complement pathway, consequently being rather anti-inflammatory than IgG antibodies [130]. The ability to oligomerize enables IgA to bind and crosslink several Fc-receptors, which was reported to release the chemoattractant LTB4, engaging neutrophils for tumor cell killing [131]. Disadvantages, such as the short serum half-life, could be overcome by antibody engineering [132].

The antibody cmHSP70.1 was also reported to be efficiently endocytosed by tumor cells, offering a specific route for targeted therapy [127]. Therefore, superparamagnetic iron oxide nanoparticles (SPIONs), a specific group of magnetic nanoparticles (MNPs), were decorated with the antibody [133,134]. The results showed an increase in retention of the particles in a glioma model in vivo [133]. Generally, MNPs can be used for diagnosis via magnetic resonance imaging or exploited for therapy by applying an alternate magnetic field (AMF) [135]. The AMF results in the spinning of iron particles, generating heat and killing the tumor by hyperthermia [135]. Interestingly, inducing ionizing radiation after injecting the particles significantly increased the retention of the particles in the tumor [134]. This is in line with the findings that radiation increases mHSP70 in tumor cells [136].

An alternative to MNPs are gold nanoparticles (AuNPs), which have a good biocompatibility and are easy to synthesize, although their costs of synthesizing are quite high [136]. They can be used for several applications, including delivering different compounds and photothermal or photodynamic therapy [136]. Recently, AuNPs coated with cmHsp70.1 mAb were shown to enable computed tomography of tumors in a preclinical study [137].

Using extracellular vesicles as specific carriers of drugs is another promising approach. Since EVs are naturally occurring vesicles, side-effects of the carrier should be minimal. Moreover, EVs could be engineered to express HSP70 and specifically target tumor cells while carrying distinct molecules for tumor cell killing. In 2010, Xie and colleagues demonstrated that mHSP70 engineered exosomes efficiently induced DC maturation, which increased T- and NK cell-dependent tumor killing [138]. Other reports showed successful loading of the chemotherapeutic paclitaxel into exosomes, resulting in tumor growth inhibition [139,140].

### 4.2. HSP70 Peptides

Since exHSP70 was shown to stimulate immune cells for tumor cell killing, the 14mer-peptide, which was used to generate the antibody, was also investigated as a therapeutic option. In the early 2000s, Multhoff and co-workers reported TKD as a recognition structure for NK cells and showed that it stimulated the proliferative and cytolytic activity [113]. Shortly after, TKD was successfully studied in a phase I trial, where NK cells were stimulated ex vivo, together with low doses of Il-2 [141]. This was supported by a recent phase II trial, where the peptide showed promising results in non-small cell lung cancer [142]. Moreover, together with the inhibition of the programmed cell death protein 1 (PD-1), a prolongation in patient survival was reported in several phase II trials of glioblastoma and lung cancer [143,144].

Another group developed a small peptide targeting the SBD of HSP70. The A8 peptide aptamer showed a decrease in MDSC and inhibited tumor progression in vivo [94]. This was due to a higher affinity of mHSP70 to A8 compared to the TLR2, thus blocking HSP70/TLR2 interaction. Therefore, A8 effectively blocked the exosome-mediated activation of MDSCs. Moreover, MDSC proliferation was also hampered since TLR2-dependent production of IL-6 via pSTAT3 was blocked. Interestingly, treating mice with cisplatin or 5-Fluorouracil led to increased HSP70-bearing exosomes, enhancing activation of MDSCs [94].

Recently, Lin and colleagues demonstrated that a 72 bp long peptide (Tx-01) could suppress ovarian cancer migration and growth in vivo [145]. They showed that Tx-01 is internalized by endo- or pinocytosis, where it binds to iHSP70, which consequently blocks the interaction of HSP70 with the Notch1 intracellular domain (NICD). This hampers the nuclear localization of HSP70, decreasing tumor invasion and migration. In addition, Tx-01 can also bind to mHSP70 leading to a rapid internalization, where it subsequently blocks the translocation of HSP70. Furthermore, the binding of Tx-01 to mHSP70 could be used as a prognostic marker in serous ovarian carcinoma [145].

In addition to peptides and antibodies, granzyme B has been reported to bind to mHSP70, leading to internalization and subsequent apoptosis of the cells [44]. Moreover, the enzyme showed significant tumor suppression of HSP70-positive colon carcinoma in vivo [31]. Recently, granzyme B-tagged SPIONs were developed, demonstrating increased survival of mice with glioblastoma or late-stage lung cancer [146]. Magnetic targeting further enhanced the localization of the SPIONs within the tumor. In addition, the nanoparticles could be used to image HSP70-positive tumors by magnetic resonance imaging [146].

### 4.3. HSP70 Vaccines

Since exHSP70 was shown to trigger T-cell responses, using it as a vaccine is another potential therapy approach. In particular, tumors are stimulated to secrete HSP70 (and antigens), which can subsequently be purified. HSP70-peptide complexes are then used as vaccines, where they mediate cross-presentation of the antigen in DCs, finally activating CD8^+^ T-cells [147]. This approach was already investigated in the early 2000s, where researchers showed inhibition of tumor growth in a prostate cancer mouse model [147]. Moreover, using HSP70-peptide complexes from the fusion of DCs and tumor cells significantly enhanced DC maturation and T-cell responses [148].

In summary, targeting exHSP70 represents an interesting theranostic approach. Since exHSP70 is uniquely expressed in tumors and not in normal cells, targeting can be used for both imaging and therapeutic approaches. Various strategies are being investigated, including peptides, antibodies, and enzymes, that bind either directly to HSP70 on tumor cells/EVs or target receptors on immune cells. The latter would mimic a vaccination and lead to activation and engagement of immune cells for tumor cell killing. Finally, vehicles such as nanoparticles or EVs can further enhance tumor imaging or treatment by carrying cytotoxic compounds. HSP70 may also be linked as a chaperone to DNA vaccines to enhance immunogenicity in humans [149]. In this study HSP70 was linked to the HPV16 E7 sequence to facilitate uptake by antigen-presenting cells and antigen presentation. HPVE7-specific T-cell responses were generally of low frequency but increased in subjects in the second and third cohorts. Tumor regression was reported in 3 out of 9 patients; however, significancy of these results needs to be confirmed in further studies.

## 5. Conclusions

Cellular stress leads to a multitude of molecular responses, guarding cells from damages. One specific response is the upregulation of the inducible HSP70, subsequently protecting cells via chaperone activities or inhibiting apoptosis. However, in cancer, HSP70 is overexpressed, leading to tumor progression and therapy resistance. Moreover, the chaperone is translocated to the extracellular milieu by several pathways, resulting in membrane-bound, EV-associated, or soluble HSP70. Hereby, exHSP70 can interact with immune cells of the tumor microenvironment, where it operates as a double-edged sword. By targeting different receptors on immune cells, it is able to either trigger pro- or anti-tumorigenic responses. Pro-tumorigenic responses include stimulating neutrophils or monocytes to secrete pro-inflammatory cytokines or activating MDSC, which results in reduced T-cell activity. In contrast, anti-tumorigenic responses comprise activation of NK cells, either directly or indirectly via DCs, and activation of T-cell by stimulating monocytes. Moreover, mediating (cross-)presentation of antigens on MHC class I or II molecules also activates the adaptive immune response for tumor cell killing. Since exHSP70 is uniquely expressed in cancer compared to normal tissue, it is a valuable therapeutic target. Therapies can include targeting exHSP70 or its receptors on tumor-, immune cells or EVs. Moreover, HSP70-antigen complexes can be used to prime immune cells for an effective anti-tumor response.

Although it is widely believed that exHSP70 can trigger opposing effects, much is still unknown or critically debated. For instance, cytokine activities of exHSP70 were questioned since this might be a result of endotoxin contamination. Moreover, the actions of HSP70-protein complexes are often overlooked but, depending on the complex, could trigger different responses. Altogether, exHSP70 can emerge as a crucial player in cancer therapy; however, a better understanding of its function is essential. Therefore, more research regarding specific mechanisms as well as novel therapeutic strategies are needed.

## Data Availability

Not applicable.
